# Chinese Herbal Formula Huo-Luo-Xiao-Ling Dan Protects against Bone Damage in Adjuvant Arthritis by Modulating the Mediators of Bone Remodeling

**DOI:** 10.1155/2013/429606

**Published:** 2013-05-16

**Authors:** Siddaraju M. Nanjundaiah, David Y.-W. Lee, Brian M. Berman, Kamal D. Moudgil

**Affiliations:** ^1^Department of Microbiology and Immunology, University of Maryland School of Medicine, HSF-1, Suite 380, 685 West Baltimore Street, Baltimore, MD 21201, USA; ^2^Mailman Research Center, McLean Hospital, Harvard Medical School, Belmont, MA 02478, USA; ^3^Center for Integrative Medicine, University of Maryland School of Medicine, East Hall, 520 West Lombard Street, Baltimore, MD 21201, USA

## Abstract

Huo-luo-xiao-ling dan (HLXL) is an herbal mixture that has long been used in traditional Chinese medicine for the treatment of rheumatoid arthritis (RA) and other inflammatory disorders. Despite the availability of potent conventionally used drugs for RA, their limited efficacy in a proportion of patients coupled with their high cost and severe adverse effects has necessitated the search for novel therapeutics for this debilitating disease. Further, the control of both inflammation and bone damage is essential for effective management of arthritis. The aim of our study was to evaluate the efficacy of HLXL against arthritic bone damage in adjuvant arthritis (AA) model of RA. Our results show that HLXL treatment suppressed inflammatory arthritis and reduced bone and cartilage damage in the joints of arthritic Lewis rats. HLXL-induced protection against bone damage was mediated primarily via inhibition of mediators of osteoclastic bone remodeling (e.g., receptor activator of nuclear factor kappa-B ligand; RANKL), skewing of RANKL/osteoprotegerin (OPG) ratio in favor of antiosteoclastic activity, reduction in the number of osteoclasts in the arthrodial joint's bone, and inhibition of cytokine production and MMP activity. Our results suggest that HLXL might offer a promising alternative/adjunct treatment for both inflammation and bone damage in RA.

## 1. Introduction

Rheumatoid arthritis (RA) is a global autoimmune disease, affecting about 1 percent of the population in USA and Europe, for example [1]. The disease is characterized by chronic inflammation of the synovial tissue in the joints [1, 2]. Uncontrolled disease leads to significant disability and deformities of the hands and feet. A variety of potent antiarthritic drugs, including biologics, have been used for the treatment of RA over the past decade or so [1, 3]. However, these conventionally used drugs have limitations. Their efficacy may be limited to a subset of patients, and their use may be associated with severe adverse reactions [3, 4]. In addition, these drugs are rather expensive. Accordingly, there is a continued search for newer therapeutic agents for RA. Natural plant products belonging to the traditional Chinese medicine (TCM) represent a diverse collection of potential therapeutic agents for a wide variety of diseases including RA [5–7]. TCM represents one of the components of complementary and alternative medicine (CAM). The popularity of CAM is gradually increasing in USA and other industrialized countries. For example, according to one survey, approximately 38 percent of adults and 12 percent of children used CAM remedies for different health needs annually [8]. Thus, there is a need to optimize the composition of a herbal CAM and to define its mechanism of action before it can be considered for further trials for the treatment of RA in the near future. The present study is aimed at fulfilling these important objectives for a TCM herbal mixture for the treatment of arthritis.

Huo-luo-xiao-ling dan (HLXL), a traditional Chinese medicine (TCM) herbal formula and its modified versions have long been used in folk medicine to treat inflammatory arthritis or joint pain, referred to as the “*Bi syndrome*” [9, 10]. The use of a combination of multiple herbs is designed to exploit the additive or synergistic activities of individual herbs, as well as to balance or neutralize the toxic effects of certain herbal components by others in the mixture [5]. In our previous studies using the rat adjuvant arthritis (AA) model of human RA [11–13], we have tested the anti-inflammatory properties of a modified version of the originally used HLXL. The treatment of Lewis rats with the modified HLXL preparation was effective in reducing inflammatory arthritis [11–13]. However, it remained to be determined whether HLXL had any effect on bone and cartilage damage associated with inflammatory arthritis. The rationale for this testing lies in the fact that certain conventionally used antiarthritic agents may efficiently suppress inflammation but not bone damage [14, 15]; the opposite may be the case for other antiarthritic agents [16–19]. Therefore, for an ideal antiarthritic agent, it is imperative that its effects are tested not only on inflammation but also on bone damage. Accordingly, the mechanism underlying the influence of HLXL on bone remodeling needed to be unraveled. The present study was aimed at filling these vital gaps.

We describe in this study the results of testing HLXL in arthritic Lewis rats. We examined and compared the arthritic paws of HLXL-treated versus vehicle-treated Lewis rats by arthritic scores, radiography, and histomorphometry. We also tested the synovial-infiltrating cells (SIC) for the production of mediators of bone remodeling such as receptor activator of nuclear factor kB ligand (RANKL), osteoprotegerin (OPG), granulocyte-macrophage colony stimulating factor (GM-CSF), osteopontin (OPN), and insulin-like growth factor (IGF). Of these, RANKL promotes osteoclastogenesis, whereas OPG serves as a decoy receptor for RANKL and thereby antagonizes the effect of RANKL. Bone damage-related cytokines (IL-1*β* and IL-18) and matrix metalloproteinases (MMPs) were also tested. Our results show that HLXL affords protection against bone and cartilage damage in the joints of arthritic rats via modulating the mediators of bone remodeling. Thus, this herbal TCM targets both inflammation and bone damage in arthritis.

## 2. Materials and Methods

### 2.1. Animals

Five- to six-week-old male Lewis (LEW/Hsd) (RT.1^l^) rats were used in this study. Rats were purchased from Harlan Sprague-Dawley (HSD) (Indianapolis, IN, USA) and then maintained in the animal care facility of the University of Maryland School of Medicine, Baltimore, MD, USA. All experimental procedures performed on these rats were in accordance with the guidelines of the Institutional Animal Care and Use Committee (IACUC).

### 2.2. Composition and Characteristics of HLXL

The herbal formula huo-luo-xiao-ling (HLXL) dan tested in this study is similar to that used in our previous studies [20–23], and it consists of a mixture of 11 well-defined herbs, namely, Ruxiang (*Boswellia carterii* Birdw.), Qianghuo (*Notopterygium incisum* Ting ex H.T. Chang), Danggui (*Angelica sinensis* (Oliv.) Diels), Chishao (*Paeonia lactiflora* Pall.), Gancao (*Glycyrrhiza uralensis* Fisch.), Yanhusuo (*Corydalis yanhusuo* W.T. Wang.), Danshen (*Salvia miltiorrhiza* Bge.), Chuanxiong (*Ligusticum chuanxiong* S.H. Qiu.), Qinjiao (*Gentiana macrophylla* Pall.), Guizhi (*Cinnamomum cassia* Presl.), and Duhuo (*Angelica pubescens* Maxim). We have previously reported in detail the methods for the preparation of HLXL, for the characteristics of its component herbs and for the assessment of its toxicity [21, 22]. The batch of HLXL used in this study was thoroughly characterized by HPLC fingerprinting as in our earlier studies [11–13, 20–23]. The HPLC profile included the peak shapes, numbers, intensities, and retention times of all individual compounds (data not shown). In addition, two marker compounds, Swertiamarin and paeoniflorin, served as references for quality control purposes.

### 2.3. Treatment of Arthritic Rats with HLXL

Lewis rats were immunized subcutaneously (s.c.) at the base of the tail with 1 mg/rat heat-killed *M. tuberculosis* H37Ra (Mtb) (Difco, Detroit, MI, USA) in 200 *μ*L of mineral oil (Sigma-Aldrich). Following the onset of arthritis, these rats were randomly divided into two groups (experimental and control). Finely powdered HLXL was suspended in water, and it was fed (2.3 g/kg) to the experimental group of rats using a gavage needle (FNC-16-3, Kant Scientific Corporation, Torrington, CT, USA) beginning on the day of onset of arthritis (d 10) and then continued up to the peak phase of AA (d 18). On the corresponding days, the control group of rats received Water (the vehicle) by gavage. All rats were examined and graded regularly for the severity of arthritis as described earlier [24, 25]. The test samples were collected from rats when the disease reached the peak phase (d 18) in controls.

### 2.4. Histological Examination of Hind Paws of Rats

The hind paws were harvested from Lewis rats on d 18 after Mtb immunization and immersed for 9 d in Cal-Ex Decalcifying solution CS510-1D (Fisher Scientific, Fair Lawn, NJ, USA). Thereafter, the paws were immersed in 70% ethanol for 5 d and then embedded in paraffin, sectioned serially using a microtome, and mounted on microscope slides. Then the sections were stained either with hematoxylin and eosin (H&E) (Histology Core, UMB) [26] or with safranin O [27, 28]. Histopathological changes in the joints like synovial hyperplasia, pannus formation, and bone damage were observed under a microscope (Nikon Eclipse E800 Microscope, Nikon Industries Inc. Melville, NY, USA) using the Spot Imaging Software (Diagnostic Instruments Inc., Sterling Heights, MI, USA) and digital images were obtained.

### 2.5. Tartrate-Resistant Acid Phosphatase (TRAP) Staining

The unstained, mounted microtome sections (as described above) were dehydrated in graded concentrations of ethanol and xylene and fixed for 2 min using 3.7% formaldehyde. The sections were washed with deionized water and were incubated in the reaction mixture (acid phosphatase, Leukocyte (TRAP) Kit, Sigma-Aldrich) at 37°C in a humid and light-protected incubator for 1 h as directed by the manufacturer. Thereafter, the sections were washed again 3 times with distilled water. Later, the sections were counter-stained with hematoxylin and observed under a microscope using the Spot Imaging Software, and digital images were obtained.

### 2.6. Bone Histomorphometry of Hind Paws

TRAP-stained hind paw sections of rats (*n* = 5 per group) were used to perform bone histomorphometry with the Osteomeasure Bone Histomorphometry system (Osteometrics, Atlanta, GA, USA) linked to a Nikon Eclipse 50i inverted microscope and a Sony CCD video camera [29]. The analyses were performed on serial transverse sections through the talus (*n* = 6). Bone volume versus total tissue volume (BV/TV), the number of osteoclasts per tissue area (N.Oc/T.Ar), active resorption per bone surface area based on the ratio of osteoclast surface/bone surface area (Oc.S/BS), and the number of osteoclasts per bone perimeter (N.Oc/B.Pm) were assessed. Histomorphometric parameters follow the recommended nomenclature of the American Society for Bone and Mineral research [30].

### 2.7. Radiographic Assessment of Arthritis in Hind Paws of Rats

The severity of AA was assessed blindly on d 18 by radiography. High-resolution digital radiography (40 kV, 12 s) of hind limbs was performed on rats under ketamine-xylazine anesthesia using a Faxitron Digital X-ray system (Faxitron X-Ray, Lincolnshire, IL, USA) [29].

### 2.8. Preparation of Synovial-Infiltrating Cells (SIC), Their Restimulation with Mtb, and Testing for Mediators of Bone Damage

SIC (total SIC) were collected by cutting open the hind paw (ankle) joints of Mtb-immunized rats on d 18 using a sterile surgical blade. These SIC were washed 3-4 times with HBSS and then were cultured in a 12-well plate using DMEM supplemented with 5% fetal bovine serum (FBS), 2 mM L-glutamine, 100 U/mL penicillin G sodium, and 100 *μ*g/mL streptomycin sulfate. The nonadherent cells were removed after 90 min by washing the culture dish with HBSS [25]. The remaining cells (adherent SIC) were restimulated for 24 h with Mtb sonicate (10 *μ*g/mL) in DMEM containing 5% FBS. Thereafter, culture supernatant was collected and tested for mediators of bone remodeling by Multiplex assay in the Cytokine Core Facility (University of Maryland, Baltimore, MD, USA) using the Luminex 100 analyzer (Luminex Corp., Austin, TX, USA). In addition, the culture supernatant was tested for matrix metalloproteinases (MMPs) as described elsewhere [27].

### 2.9. Statistical Analysis

The data were expressed as mean ± SEM. Student's *t*-test and ANOVA Bonferroni's post hoc method were used to assess the significance of differences using GraphPad Prism version 4.0. A *P* value of <0.05 was considered statistically significant.

## 3. Results

### 3.1. HLXL Suppresses Inflammation and Tissue Damage in the Joints of Arthritic Rats

**Figure 1:**
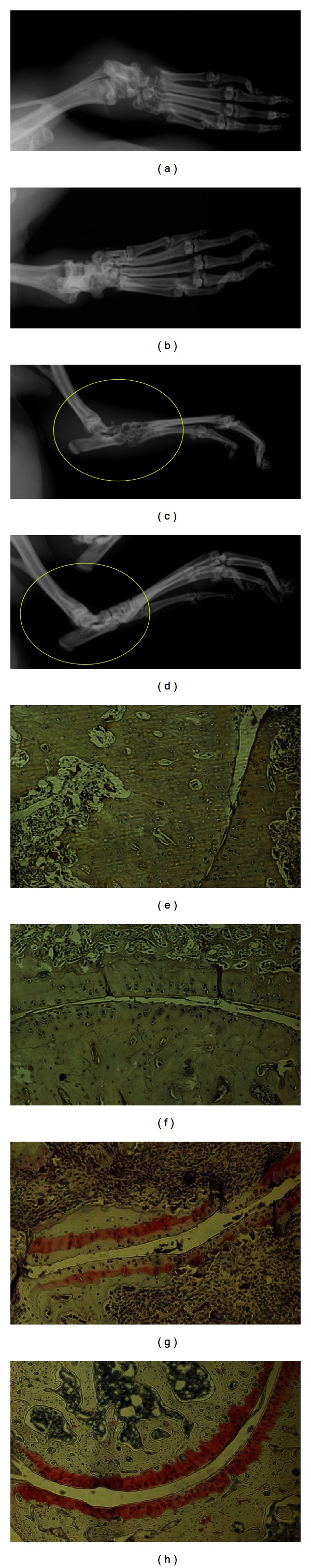
HLXL attenuates arthritic inflammation and joint damage in Lewis rats. A group each (*n* = 4 per group) of Mtb-immunized Lewis rats was fed either Water (Vehicle; (a), (c), (e), (g)) or HLXL (2.3 g/kg; (b), (d), (f), (h)), beginning at the onset of AA and then continued through d 18 after Mtb injection. (a), (b) (frontal view) and (c), (d) (lateral view) depict representative radiographs of hind limbs on d 18. Representative micrographs of tartrate-resistant-acid-phosphatase- (TRAP-) stained ((e), (f)) and safranin O-stained ((g), (h)) sections of the hind paw joints of control and HLXL-treated rats are shown.

Arthritic Lewis rats were fed daily with HLXL (in water, by gavage) beginning at the onset (d 10) of AA and then continued up to the peak phase (d 18) of the disease, whereas the corresponding control rats received water by gavage. There was a significant reduction in the severity of clinical arthritis (see Figure S1(a) in Supplementary Material available online at http://dx.doi.org/10.1155/2013/429606). Histological examination revealed significant reduction of pannus formation, synovial mononuclear cell infiltration, and bone destruction in HLXL-treated rats compared to control rats (Supplementary Figures 1(b)– 1(d)). In parallel, histological sections subjected to TRAP staining (for osteoclasts) (Figures 1(e) and 1(f)) and safranin-O staining (for cartilage) (Figures 1(g) and 1(h)) showed reduction in osteoclasts as well as cartilage damage. On d 18, the hind paws of rats were subjected to radiological examination; the radiographs showed reduction in the inflamed soft tissue around the joints and bone damage in HLXL-treated rats compared to control rats (Figures 1(a)–1(d)).

### 3.2. HLXL Suppresses Bone Loss and Osteoclast Number in Arthritic Lewis Rats

**Figure 2:**
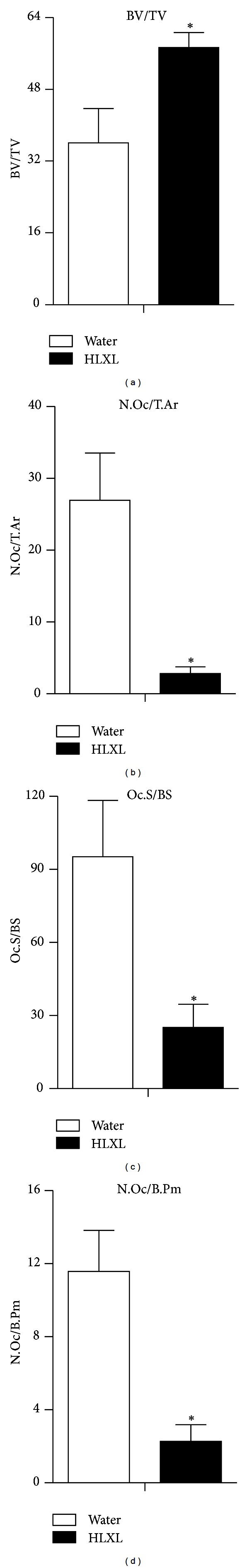
HLXL reduces bone resorption and osteoclast numbers in arthritic rats. Bone histomorphometry was performed on TRAP-stained sections of hind paws of HLXL-treated versus Water-treated rats (*n* = 5 per group). The measurements of the following parameters are shown in the figure: (a) bone volume versus total tissue volume (BV/TV); (b) the number of osteoclasts per tissue area (N.Oc/T.Ar); (c) active resorption per bone surface area based on the ratio of osteoclast surface/bone surface area (Oc.S/BS); and (d) the number of osteoclasts per bone perimeter (N.Oc/B.Pm). (*P < 0.05, comparing experimental and control samples.)

AA is characterized by bone resorption, which is evident from histomorphometric examination of hind paw sections of arthritic rats compared with those of naïve rats (Supplementary Figure 2). Therefore, we tested whether HLXL treatment altered these histomorphometric parameters. For this, subchondral bone loss and osteoclast numbers in the talus of the hind paw joints of the experimental and control rats were analyzed. We observed a significant reduction in the subchondral bone loss and increased bone volume in HLXL-treated rats compared to the control rats (Figure 2(a)). Histomorphometric analysis of tartrate-resistant-acid-phosphatase- (TRAP-) stained joint sections revealed that the number of osteoclasts (Figure 2(b)) and the corresponding active resorption surfaces (Figure 2(c)) were reduced in HLXL-treated rats compared to those of control rats. Osteoclast number/bone perimeter was also reduced after treatment with HLXL when compared to controls (Figure 2(d)).

### 3.3. HLXL Regulates Mediators of Bone Remodeling in Arthritic Rats

**Figure 3:**
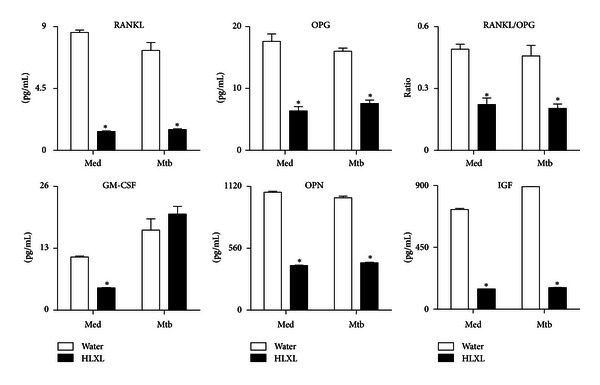
HLXL regulates mediators of bone remodeling in the joints of arthritic rats. Synovial-infiltrating cells (SIC) were harvested on d 18 from Mtb-immunized rats (*n* = 4 per group) treated with HLXL or Water (Vehicle) as described in Figure 1, and then these cells were restimulated for 24 h with Mtb sonicate (10 *μ*g/mL). The levels of the indicated mediators were measured in culture supernatants of SIC using a Multiplex assay and the results were expressed as pg/mL. *P < 0.05, comparing experimental and control samples. (RANKL: receptor activator of nuclear factor kappa-B ligand; OPG: osteoprotegerin; GM-CSF: granulocyte-macrophage colony-stimulating factor; IGF: insulin-like growth factor; OPN: osteopontin; Med: medium; Mtb: heat-killed *M. tuberculosis* H37Ra.)

To determine the mechanisms underlying the observed effects of HLXL on bone remodeling, we tested the effect of HLXL on the mediators of bone remodeling (RANKL, OPG, GM-CSF, OPN, and IGF) in experimental and control rats. These mediators were measured in culture supernatants of synovium-infiltrating cells (SIC), which had been restimulated with sonicated, heat-killed *M. tuberculosis* H37Ra (Mtb sonicate) (Figure 3). There was a significant decrease in all of the above-mentioned bone remodeling mediators tested in SIC of HLXL-treated rats compared to those of control rats (Figure 3). Though both RANKL and OPG levels were reduced but to different extents, leading to deviation of the RANKL/OPG ratio in favor of antiosteoclastic activity in HLXL-treated rats compared to control rats.

### 3.4. HLXL Treatment Inhibits Antigen-Induced Proinflammatory Cytokine Response and MMP Activity in Arthritic Rats

**Figure 4:**
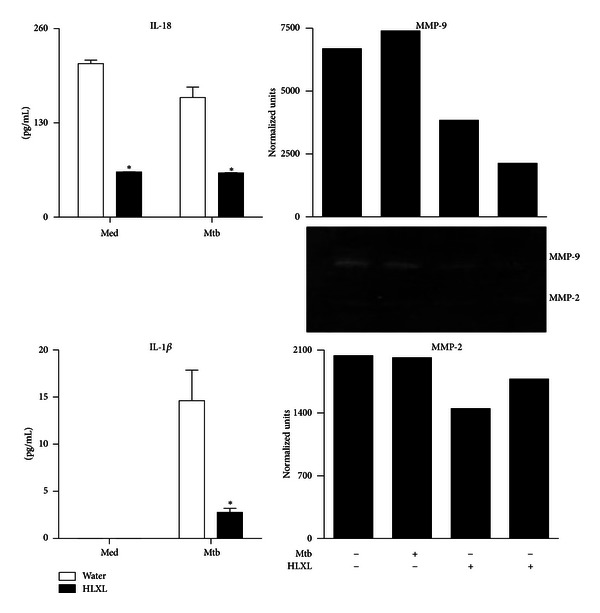
Inhibition of proinflammatory cytokines and MMP activity in HLXL-treated arthritic rats. Synovial-infiltrating cells (SIC) harvested on d 18 (peak phase of arthritis) from HLXL-fed or Water-fed rats (*n* = 4 per group) were cultured for 24 h with or without sonicated Mtb. The levels of cytokines IL-18 and IL-1*β* (left panel) in culture supernatants were measured using a Multiplex assay and the results were expressed as pg/mL. MMP-9 and MMP-2 activity (right panel) was tested in the culture supernatants of Mtb-restimulated SIC cells of HLXL/Water-treated rats. Mtb: heat-killed *M. tuberculosis* H37Ra; Med: medium. (*P < 0.05, comparing experimental and control samples.)

IL-18 and IL-1*β* are proinflammatory cytokines that have a significant effect on bone remodeling. Therefore, we tested the levels of these two cytokines (as proteins) in SIC that were harvested from HLXL-treated and control arthritic rats and then restimulated *in vitro* for 24 h with sonicated Mtb. There was a significant decrease (*P* < 0.05) in the level of IL-18 as well as IL-1*β* in HLXL-treated rats compared with Water-treated rats (Figure 4, left panel). We also tested in SIC the levels of MMPs (MMP-2 and MMP-9), one of the key mediators of tissue damage in arthritis (Figure 4, right panel). The levels of these MMPs were reduced in HLXL-treated rats compared to controls.

## 4. Discussion

Herbal TCM and other CAM modalities of the traditional systems of medicine have long been used for the treatment of RA and other inflammatory disorders in different parts of the world [31–34]. The use and popularity of CAM products have gradually been increasing in the western countries [35, 36]. In this context, it is imperative that the composition of any herbal CAM to be considered for arthritis therapy is documented adequately. In addition, it is essential to define the mechanisms of action of herbal CAM to bring them to the mainstream of therapeutic arsenal for RA and other diseases. These priorities are essential to meet if herbal CAM is to be used as an adjunct to or in lieu of conventionally used drugs for RA. In a previous study, we have described the HPLC profile of HLXL and identified various compounds isolated, including steroids, terpenes, alkaloids, flavonoids, glycosides, and acids [23]. In another set of studies, we have elaborated the anti-inflammatory properties of HLXL [11, 13]. In this study, we have taken the first steps to fulfill above obligations regarding the use of HLXL, a Chinese herbal mixture, for the treatment of experimental arthritis in Lewis rats.

Our results show that treatment of arthritic Lewis rats with HLXL significantly reduced inflammation of hind paws as assessed by arthritic scores. This effect was further confirmed by histopathological examination of hind paw joints. Importantly, HLXL treatment also afforded protection against bone and cartilage damage. This was validated by histopathological and radiological examination of hind paws and finally confirmed by histomorphometry, which showed that HLXL-treated rats had higher bone mass, reduced bone resorption, and increased number of osteoclasts in the tissue section observed compared to control (Water-treated) rats. Thus, HLXL was effective in reducing both inflammation and bone damage in arthritic joints.

Bone remodeling is a balance of bone-forming (osteoblastic) and bone-resorping (osteoclastic) activities [37, 38]. Osteoclast-regulated bone remodeling is critically dependent on the activity of RANKL-RANK-OPG axis. RANKL is a tumor necrosis factor ligand superfamily member, and it is produced by osteoblasts [39]. RANKL binds to its cognate receptor RANK, which is expressed on osteoclast progenitors, mature osteoclasts, and chondrocytes [40–42]. The expression of RANKL can be induced by proinflammatory cytokines such as TNF-*α*, IL-1*β*, IL-6, and IL-17. RANKL acting with M-CSF is critical for the process of osteoclastogenesis and it influences the activation, maturation, and survival of osteoclasts. M-CSF is mainly produced by mature osteoblasts and it binds to colony-stimulating factor 1 receptor (c-fms) expressed on the surface of osteoclast precursors [43]. Other cellular sources of M-CSF are chondrocytes and synovial fibroblasts. IL-1 and TNF-*α* promote M-CSF production. OPG, also known as osteoclastogenesis inhibitory factor (OCIF), is a soluble protein that serves as a decoy receptor for RANKL [44, 45]. OPG competes with RANK for binding to RANKL, and it inhibits the maturation and activation of osteoclasts [39]. Soluble RANKL may not be a good indicator of bone loss as most RANKL is membrane bound. Interestingly, our results showed that HLXL-treated rats had a deviation of the RANKL/OPG ratio in favor of antiosteoclastic activity compared to Water-treated rats.

**Figure 5:**
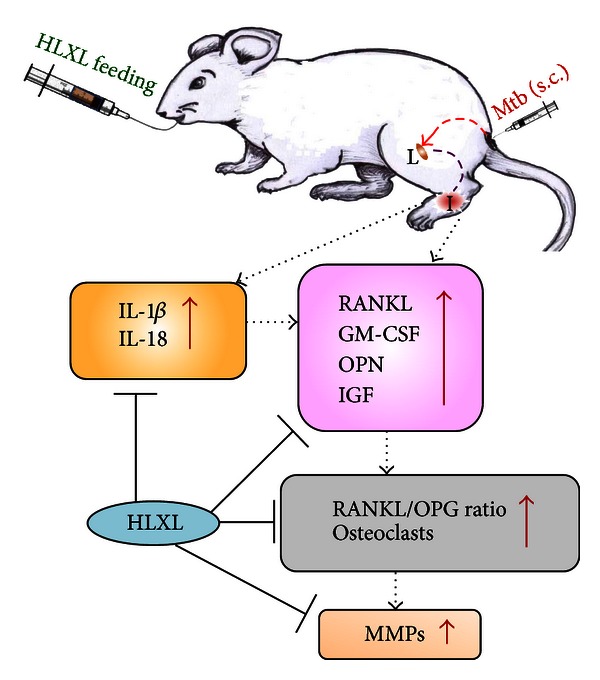
A schematic representation of the mechanisms involved in HLXL-induced suppression of bone damage in AA. HLXL suppressed bone damage via inhibiting the expression of osteoclastogenic mediators (receptor activator of nuclear factor kappa-B ligand: RANKL; granulocyte-macrophage colony-stimulating factor: GM-CSF; osteopontin: OPN; and insulin-like growth factor: IGF), cytokines (IL-18 and IL-1*β*), and matrix metalloproteinases (MMPs). (L: lymph node, I: inflamed joint, Mtb: heat-killed* Mycobacterium tuberculosis* H37Ra, osteoprotegerin: OPG.) Red arrows highlight increased amount/activity of the indicated mediators in arthritis, which are inhibited by HLXL treatment.

Additional mediators of bone remodeling include GM-CSF, OPN, and IGF [46–48]. GM-CSF is produced by a variety of cells including macrophages, T cells, endothelial cells, and fibroblasts. It regulates the fusion of mononuclear osteoclasts into bone-resorbing osteoclasts [49]. OPN is produced by synovial fibroblasts, and it facilitates osteoclastic activity while suppressing osteoblastic activity [48]. In addition, OPN can enhance angiogenesis [50] as well as production of proinflammatory cytokines IL-6 and IL-17 [51, 52]. IGF mediates bone and cartilage degradation. Increased amounts of IGF are present in the synovial fluid of RA patients [47]. Interestingly, HLXL treatment significantly reduced the levels of GM-CSF, OPN, and IGF. Taken together with the results of altered RANKL/OPG ratio, these results show that HLXL treatment modulated the levels of the key mediators of bone remodeling (Figure 5).

The proinflammatory cytokines are among the vital inducers of some of the mediators of bone remodeling. Prominent among these cytokines are TNF-*α*, IL-1*β*, IL-6, IL-17, and IL-18. In this study, we showed that HLXL treatment significantly reduced the production of IL-1*β* and IL-18 by synovial-infiltrating cells compared to Vehicle (Water) treatment. In our previous study using HLXL, we showed that HLXL reduced the production of IL-6 and IL-17 [11]. However, in that study, we had measured the effect of HLXL on inflammation component of arthritis but not on the bone remodeling parameters. The present study has filled that gap. Further, our results on MMP testing in HLXL-treated rats in SIC are supported by our earlier finding of HLXL-induced reduction in MMPs in spleen adherent cells (SAC) of arthritic rats [11]. Taken together, the results of our present and previous studies show that HLXL has a prominent effect on bone remodeling in part via reducing the production of various proinflammatory cytokines and MMPs (Figure 5).

## 5. Conclusion

In summary, our results demonstrate the antiarthritic activity of a Chinese herbal mixture, HLXL. Realizing the significance of properly documenting the composition of an herbal CAM, we performed our study using a well-characterized herbal mixture, HLXL. Further, to enhance the confidence of the public as well as the professionals in the rational use of a herbal mixture, we have invested effort in examining the mechanism by which HLXL protects against bone damage in arthritic joints (Figure 5). On the basis of the results of our study reported here combined with those of our previous studies on HLXL, we conclude that HLXL targets both inflammation and bone damage components of autoimmune arthritis, and that HLXL should be further evaluated in a preclinical study in RA patients.

## Supplementary Material

HLXL-treatment leads to reduction in the severity of arthritis as tested in the rat adjuvant arthritis (AA) model of human rheumatoid arthritis. Experimental group of arthritic Lewis rats was treated with HLXL, whereas the control group of arthritic rats was treated with the vehicle (Water). The treatment was begun at the onset of AA and then continued daily through the peak phase of AA. The severity of arthritis was assessed by arthritic scores and histological examination (Supplementary Figure 1) as well as by bone histomorphometry (Supplementary Figure 2) of hind paws.
