# Supplementary figures and images for: Chinese Herbal Formula Huo-Luo-Xiao-Ling Dan Protects against Bone Damage in Adjuvant Arthritis by Modulating the Mediators of Bone Remodeling
Source: Evid Based Complement Alternat Med. 2013 May 16;2013:429606. doi: 10.1155/2013/429606 (PMC3670518; doi:10.1155/2013/429606)

Supplementary Fig 1

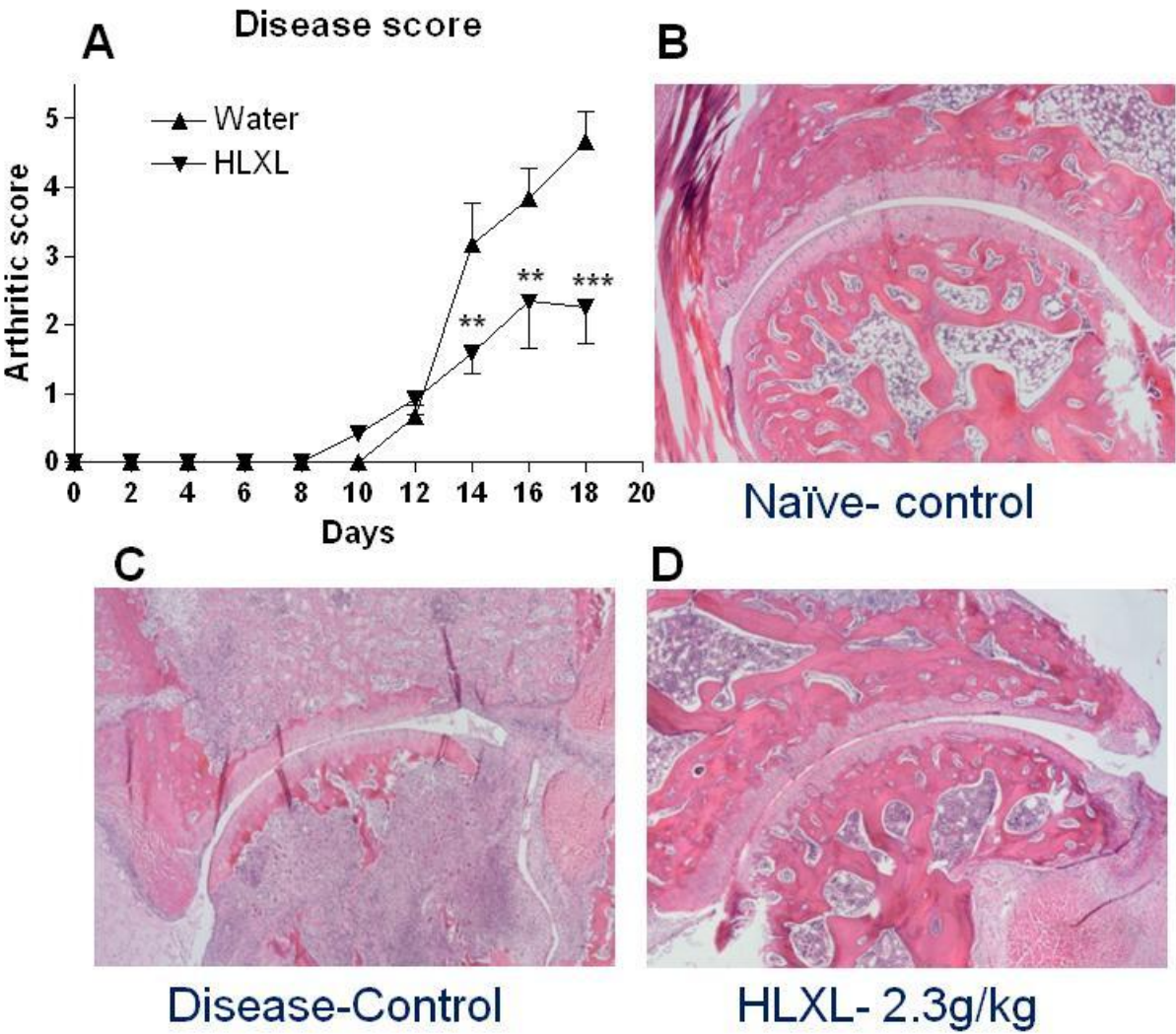

Supplementary Figure 2

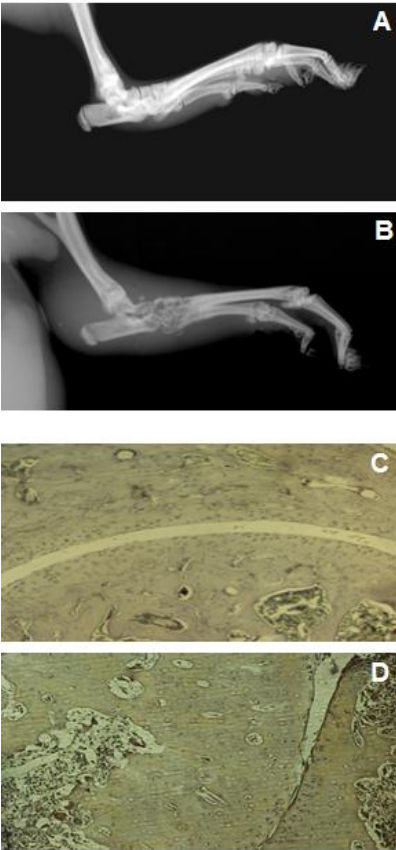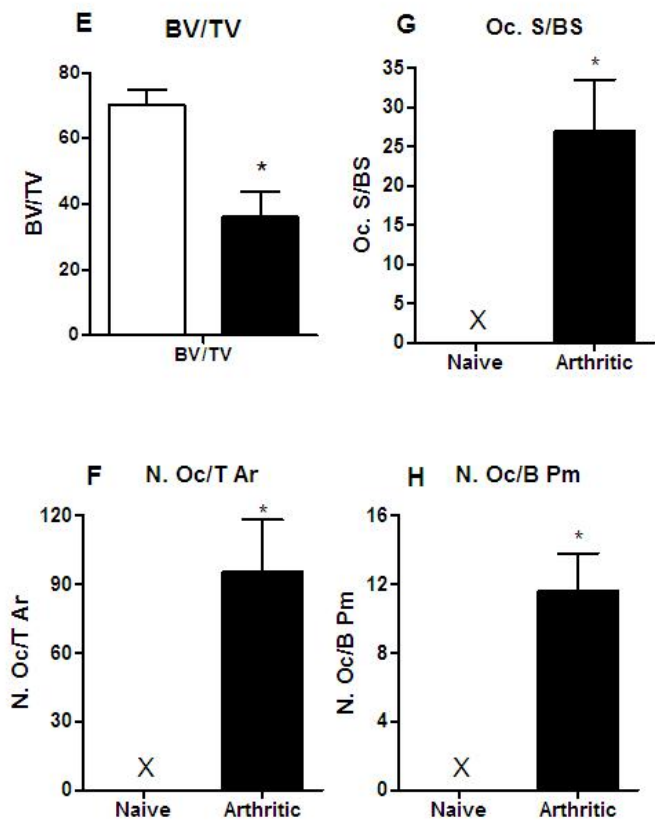

Supplement: Supplementary file 1 — HLXL-treatment leads to reduction in the severity of arthritis as tested in the rat adjuvant arthritis (AA) model of human rheumatoid arthritis. Experimental group of arthritic Lewis rats was treated with HLXL, whereas the control group of arthritic rats was treated with the vehicle (Water). The treatment was begun at the onset of AA and then continued daily through the peak phase of AA. The severity of arthritis was assessed by arthritic scores and histological examination (Supplementary Figure 1) as well as by bone histomorphometry (Supplementary Figure 2) of hind paws. [file 429606.f1.pdf]
